# Practical Considerations for Laser-Induced Graphene Pressure Sensors Used in Marine Applications

**DOI:** 10.3390/s23229044

**Published:** 2023-11-08

**Authors:** Tessa Van Volkenburg, Daniel Ayoub, Andrea Alemán Reyes, Zhiyong Xia, Leslie Hamilton

**Affiliations:** 1 Research and Exploratory Development Department, Johns Hopkins University Applied Physics Laboratory, Laurel, MD 20723, USA; tessa.vanvolkenburg@jhuapl.edu (T.V.V.); daniel.ayoub@jhuapl.edu (D.A.); zhiyong.xia@ghd.com (Z.X.); 2 Department of Chemical Engineering, University of Puerto Rico at Mayaguez, Mayaguez 00681, Puerto Rico; andrea.aleman@upr.edu

**Keywords:** laser-induced graphene, piezoresistive, pressure sensor

## Abstract

Small, low-power, and inexpensive marine depth sensors are of interest for a myriad of applications from maritime security to environmental monitoring. Recently, laser-induced graphene (LIG) piezoresistive pressure sensors have been proposed given their rapid fabrication and large dynamic range. In this work, the practicality of LIG integration into fieldable deep ocean (1 km) depth sensors in bulk is explored. Initially, a design of experiments (DOEs) approach evaluated laser engraver fabrication parameters such as line length, line width, laser speed, and laser power on resultant resistances of LIG traces. Next, uniaxial compression and thermal testing at relevant ocean pressures up to 10.3 MPa and temperatures between 0 and 25 °C evaluated the piezoresistive response of replicate sensors and determined the individual characterization of each, which is necessary. Additionally, bare LIG sensors showed larger resistance changes with temperature (ΔR ≈ 30 kΩ) than pressure (ΔR ≈ 1–15 kΩ), indicating that conformal coatings are required to both thermally insulate and electrically isolate traces from surrounding seawater. Sensors encapsulated with two dip-coated layers of 5 wt% polydimethylsiloxane (PDMS) silicone and submerged in water baths from 0 to 25 °C showed significant thermal dampening (ΔR ≈ 0.3 kΩ), indicating a path forward for the continued development of LIG/PDMS composite structures. This work presents both the promises and limitations of LIG piezoresistive depth sensors and recommends further research to validate this platform for global deployment.

## 1. Introduction

Accurate and repeatable in-situ ocean depth sensing is essential for a variety of fields, from tracking endangered marine mammals to wave detection for maritime security [1] to mapping the impact of climate change [2]. Sampling campaigns will often leverage existing sensor platforms such as drifters that measure the ocean surface conditions, moored profilers and profiling floats that sample the water column, unmanned surface vehicles (USVs) for ocean/air boundary conditions, and autonomous underwater vehicles or tagged marine mammals for deep ocean measurements [3,4,5,6,7]. Recently, researchers have emphasized the need for increased spatial and temporal resolution to accurately predict ocean changes, but such coverage is difficult and expensive to implement on a grand scale [8]. Most deployed conductivity, temperature, and depth (CTD) sensors are made from rigid materials (i.e., titanium alloys) capable of withstanding high hydrostatic pressures and necessitating watertight seals to protect supporting electronics [6]. Depending on the depth, logging capability, and sensitivity, high-end commercial compact CTD sensors can range from several hundred to several thousand dollars per sensor, which prohibits high-volume deployment. Therefore, flexible, rapidly manufactured, and cost-effective sensing platforms that approach the accuracy and sensitivity requirements of currently fielded devices could revolutionize marine monitoring.

A particular challenge is creating sensors capable of withstanding ocean depth up to one kilometer, the range where most variability of oceanic processes is seen [9], without sacrificing measurement sensitivity. Additionally, pressure measurements must be accurate to within ≤3 dbar (0.3 MPa) to correctly describe physical seawater measurements such as density, temperature, and salinity within the water column [10]. Similarly, external calibration against reference standards and on-board compensation must be included to account for factors that could impede resistive measurements like thermal fluctuations and marine biofouling. Current autonomous sampling platforms emphasize the need for technologies with fast dynamic responses to changing environments and stability during long deployments. In both commercially available and state-of-the-art CTD sensors, most depth measurements are classified as resonant quartz crystal sensors, optical fiber sensors, or piezoresistive sensors [11,12]. Piezoresistive sensors constitute the majority of cataloged sensors, exhibit a fast resistance change in response to mechanical load, and have a simpler working mechanism that requires less expensive fabrication.

Recently, laser-induced graphene (LIG) sensors have emerged as one of the most promising piezoresistive sensors given their low cost and rapid manufacturability. First described in 2014 [13], LIG films have been demonstrated with numerous substrates (polyimide, paper, etc.) for sensing applications in healthcare, environmental monitoring, energy harvesting, robotics, and more [14,15,16]. LIG is directly patterned on a carbon-containing substrate (typically polyimide, commercially known as Kapton^®^) by using a high-powered carbon dioxide (CO_2_) laser. The laser energy breaks carbon and oxygen single and double bonds and carbon nitrogen bonds at the surface. These then rapidly reform to create a “kinetic graphene” characterized by five and seven membered graphene rings in addition to the usual six. The gasses released from this process also cause LIG’s characteristic pores [17]. The presence of strained rings and pores creates a resistive layer with typical resistivity values in the range of 1 × 10^−3^–7 × 10^−3^ Ω·m depending on the fabrication parameters used [18].

LIG sensors have also been previously proposed as deep ocean CTD sensors due to their tolerance to extreme marine pressures and cold temperatures. Recent work by Kaidarova et al. tested the sensor in a compression chamber at depths up to two kilometers, with relative resistance changes corresponding to sensitivities of 2 × 10^−5^ kPa^−1^ from 0 to ~6 MPa and 0.2 × 10^−5^ kPa^−1^ from ~6 to ~12 MPa [19]. The authors note that the resistance change is smaller at higher pressures due to smaller changes in the relative resistance as the pores are compressed, indicating that there is an upper limit to the pressure range sensed. This work demonstrated the feasibility of LIG traces as marine pressure sensors, but questions still exist about the effect of LIG fabrication and resultant morphology on the sensitivity range, robustness of these films over time, and the repeatability of the complete sensor fabrication process. For example, LIG films are notoriously tricky to replicate, and the quality of graphene formed depends on the laser power, speed, beam size, focal height, atmosphere, and substrate. Previous work has also shown that high-laser-fluence LIG films have different fibrous surface morphologies than lower-laser-fluence films [20]. This morphological change can impede good electrical connections with the LIG films and affect the overall sensor performance. Large LIG surface areas are near impossible to wet with traditional solders without surface modification, high pressure, or high temperature [21,22], and the high carbon–carbon bonding ratio inhibits metallurgical connection [21]. Additionally, LIG traces have been previously used as thermistors at similar sensitivities for marine environments, indicating a temperature dependence on LIG resistivity [19]. In this paper, we include studies that investigate the impact of laser fluence on LIG surface morphology and resistivity, the repeatability of fabrication and response with uniaxial compression testing, and the impact of thermal response with LIG sensors before and after conformal coatings. Recommendations for future work and the limitations of LIG as marine sensors are also discussed.

## 2. Materials and Methods

### 2.1. LIG Sensor Fabrication

Polyimide (Kapton^®^, DuPont, Wilmington, DE, USA) films with silicone adhesive backing were purchased from Grainger (Lake Forest, IL, USA). Copper tape, Kapton^®^ tape, silver epoxy, and stainless-steel wire were all purchased from DigiKey (Thief River Falls, MN, USA). All LIG films were fabricated using the CO_2_ head of the Fusion M2 50W dual CO_2_ and fiber laser (Epilog Laser, Golden, CO, USA) using laser powers between 45 and 60% of the total laser power, and speeds between 55 and 70% of the total speed. The exact parameters are discussed further in Section 3.1 LIG Characterization. Linear and serpentine patterns were made in CorelDRAW (Version 23, Ottawa, ON, Canada).

### 2.2. SEM Imaging

A Thermo Fisher Scientific™ Scios™ (Waltham, MA, USA) scanning electron microscope (SEM) was used to record changes in the morphology of the LIG sensors before and after compression testing. A relatively small acceleration voltage of 5 kV was used to limit the penetration of the beam into the material in order to capture morphological information closer to the surface of the samples. Images were taken in a cross-section using a razor blade to cleanly slice traces to observe the thickness of the top layer, and they were also taken in the top-down orientation to determine the porosity of the hierarchical structures.

### 2.3. Compression Testing

Uniaxial compression testing was performed on a 6800 Series Universal Testing System (Instron, Norwood, MA, USA) with a 300 kN load frame and 50 N load cell. The experimental setup is further described in Section 3.2. Sensors were sandwiched between two steel blocks (20 mm × 20 mm × 13 mm) to protect the load cell during the test due to the small displacement of the LIG sensors. The resistance of the sensor was measured using a voltage divider connected to a multifunction I/O device (National Instruments, Austin, TX, USA). Voltage values for resistance (*V_sens_*), load, and displacement were recorded every 0.2 s using DAQ Express software (National Instruments Version 5.1), and sensor resistance was calculated (*R_sens_*) based on Equation (1) for a voltage divider with 5 VDC input (*V_in_*) (Kiethley, Cleveland, OH, USA) and a 10 kΩ reference resistor (*R_ref_*). During the test, the sensors were (1) loaded at a constant displacement (0.25 mm/min) up to 10.3 MPa (~1030 m ocean depth), (2) held for one minute, (3) unloaded at the same displacement to 6.9 MPa (~687 m ocean depth), (4) held for another minute, and then (5) unloaded to ~0.3 MPa. Subsequent presses were repeated in triplicate before removing the sensor from the load frame.

(1) $$R_{sens}=\frac{V_{in}\times R_{ref}}{V_{in}-V_{sens}}$$

### 2.4. Thermal Testing

To determine the impact of combined thermal and compression testing, a Materials Test Systems (MTS) load frame with environmental chamber (MET Laboratories Inc., Baltimore, MD, USA) cooled via liquid nitrogen was used. Compression measurements were performed using the same experimental setup as uniaxial compression testing, except the resistance and source voltage were now measured directly across the sensor with a Kiethley Sourcemeter using the same load profile (0.25 mm/min displacement to 10.3 MPa, hold 1 min, 0.25 mm/min unload to 6.9 MPa, hold 1 min, unload to ~0.3 MPa). Each sample tested was allowed to equilibrate to the surrounding temperature (0 °C, 15 °C, and 25 °C) for five minutes before beginning the load profile.

Prior to aqueous thermal testing, sensors were given conformal coatings to both improve the mechanical robustness of the LIG layer and electrically isolate from water. Four different coatings were prepared, including (1) a thick (~5 mm) layer of 10 wt% (curing agent in base) polydimethylsiloxane (PDMS) silicone, (2) a single dip-coated layer of 5 wt% PDMS silicone, (3) two dip-coated layers of 5 wt% PDMS silicone, and (4) a thin (0.1 mm) layer of parylene-c. The PDMS silicone (SYLGARD^TM^ 184, Dow, Midland, MI, USA) was prepared and degassed according to the manufacturer’s instructions. To speed up curing between dip-coated layers, sensors were cured in an oven at 60 °C. Parylene-c layers were vapor deposited using a custom chamber. 

The experimental setup used for the benchtop thermal testing is shown in Section 3.3. A 250 mL jacketed beaker (Cole Parmer, Vernon Hills, IL, USA) was filled with tap water and the sensor was placed inside. A chiller was connected to the inlet and outlet of the jacket, allowing coolant to chill the beaker from room temperature to 1 °C in approximately 20 min. The temperature of the tap water was recorded with a logging thermocouple every one second with an integrated python script. The resistance of the sensor was measured using the voltage divider setup mentioned previously for uniaxial testing. Between samples, the tap water was refreshed, and the coolant was returned to room temperature.

## 3. Results

### 3.1. LIG Characterization

Exemplar LIG structures were printed on polyimide films with an adhesive backing layer using a CO_2_ laser engraver. Initial prints determined the laser parameters that formed good resistive LIG structures, including minimum linewidth (0.5 mm) and spacing (0.7 mm) that retained sufficient linewidth resolution (Figure S1 and Table S1). Similarly, a design of experiments (DOE) determined effective speed and power combinations, or recipes, for LIG formation. The DOE method uses statistical analysis to allow the user to simultaneously change multiple input values (factors) and evaluate the significant individual and combinatorial effects on the key output variables (responses) in complex design spaces more rapidly than one factor at a time (OFAT) analysis. The DOE evaluated print completeness and LIG resistivity by changing four input factors: laser power (50%, 55%, 60%, and 65% of the total 50 W CO_2_ laser), print head speed (55%, 60%, 65%, and 70% of the total 419 cm/s laser head), specified line width (0.5, 1.0, and 1.5 mm), and specified line length (11.8–118 mm). An ohmmeter evaluated each print’s resistance (kΩ) across contact patches, and incomplete prints were not included in the analysis. John Macintosh’s Project (JMP) statistical analysis software (Version 17) analyzed the results and determined that laser speed and power are the most important factors to predict print completeness, while line length and power are the most important factors to predict overall resistance (see Figure S2 and Table S2 for full analysis).

The DOE results identified four LIG recipes suitable for further testing. Table 1 shows each recipe’s power and speed percentages, and the approximate laser fluence (*H*), or energy per area (J/cm^2^), used to create each (Equation (2)) [20,23]. Here, speed (*S*) and power (*P*) are represented as percentages of the maximum for the laser engraver; A is the laser beam area (beam diameter 76.2 µm) [24], and PPI (pulses per inch) is the pulse density of the laser (1000 *PPI*). It is important to note that the laser fluence estimates are approximate values since the print head speed percentage does not scale linearly [25]. Additionally, bounding boxes printed around each active area attempted to prevent local acceleration effects as the laser head traversed the print bed.

(2) $$H=\frac{P}{S\times A\times PPI\;}$$

**Figure 1:**
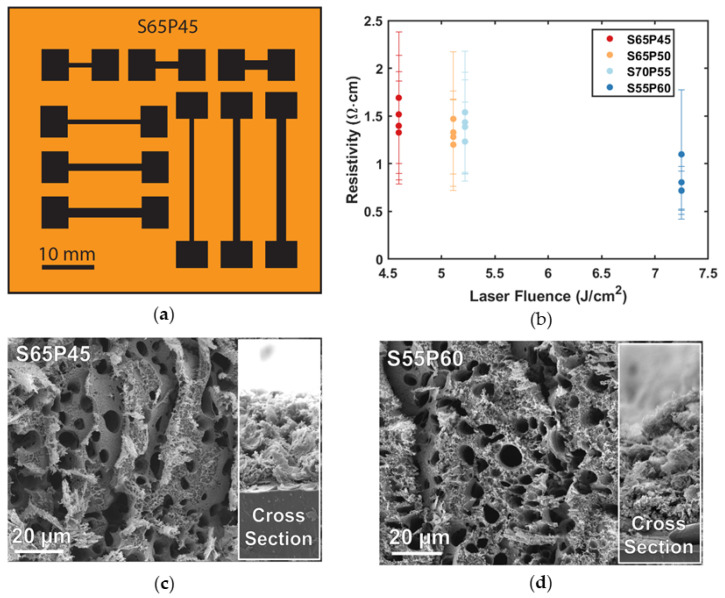
Testing repeatability of LIG recipe (S = % laser speed, P = % laser power) fabrication. (**a**) Schematic of a test swatch with different line lengths (5, 10, and 15 mm) and widths (1, 2, and 3 mm), enabling the calculation of average resistivity for each recipe. (**b**) Comparison of laser resistivity with each on separate test swatches (print bed re-leveled each time). SEM images of LIG samples with top-down and cross-sectional (inset) views (**c**) the lowest laser fluence (S65P45) and (**d**) the highest laser fluence (S55P60).

With four recipes identified, multiple laser prints determined the repeatability of LIG trace formation and resistance (*R*). Figure 1a shows the printed test swatches where line lengths (***L***) and widths (*W*) were varied to account for within-print variability in resistivity (*⍴*). Additionally, subsequent laser engraver prints, where the laser head was power cycled and print bed re-focused between prints, allowed for a characterization of the between-print variability to be conducted. Figure 1b compares the between-print variability (distinct dots) and within-print variability (error bars) for different print recipes (different colors). SEM images show negligible differences in LIG thickness (*t*~50 µm) with increasing laser fluence (Figure 1c,d), allowing Equation (3) to calculate the resistivity of each trace. Additionally, the latter figures show LIG surface porosity of the lowest (Figure 1c) and highest (Figure 1d) laser fluence recipes. Additional SEM images of other recipes are included in Figure S3.

(3) $$\rho =\frac{R\times W\times t}{L}$$

The characteristic porous morphology of the SEM images and the relative resistivity values in Figure 1 indicate that LIG traces are being formed. However, although in general LIG resistivity decreases with increasing laser fluence (different colors), the within-print variability is greater (seen by overlapping error bars). Essentially, an LIG recipe cannot be used to accurately predict LIG resistivity and thus overall sensor resistance; every sensor must be individually characterized. Next, load frame testing evaluated LIG piezoresistive performance by integrating into complete sensors.

### 3.2. Uniaxial Compression Testing

**Figure 2:**
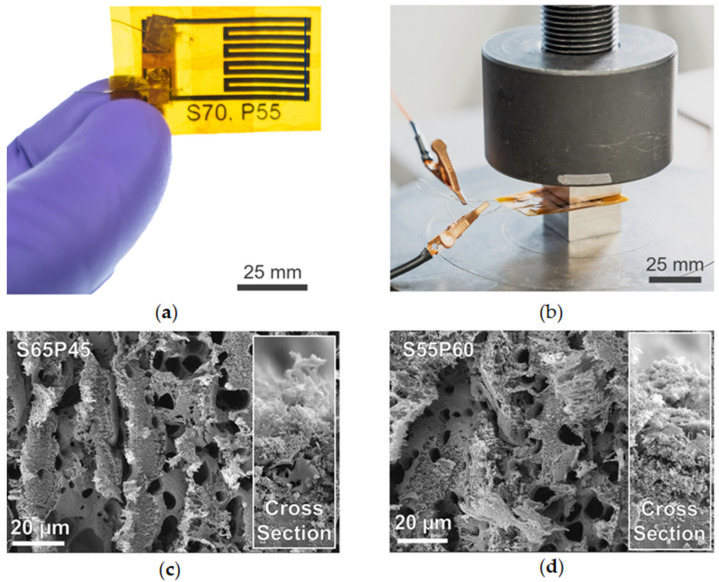
Instron compression testing of LIG sensors during repeated presses. LIG traces were assembled into (**a**) LIG piezoresistive sensors for testing using the (**b**) experimental setup of sensor compressed between two steel blocks. Recovery of LIG porous structure is shown after six cycles for (**c**) the lowest laser fluence and (**d**) highest laser fluence.

To evaluate the piezoresistive performance of LIG sensors, stainless-steel wires were attached to printed contact patches with silver epoxy and copper tape. Figure 2a shows the final sensor with an active area measuring approximately 20 mm × 30 mm. The longer dimension on one side ensured that contact patch connections were not compressed during testing. Figure 2b illustrates the compression scheme of sensors sandwiched between two 13 mm thick steel blocks to protect the load cell during compression with thin Kapton sheets (~200 µm). A smaller piece of Kapton tape secured the lead wires in place, but the LIG surface remained uncoated. To prevent shortening with the steel blocks, another piece of Kapton tape coated the underside of the top steel block. Figure 2c,d show the SEM images of the LIG surface and cross-section after six successive presses to 10.3 MPa, demonstrating the recovery of the porous structure. 

**Figure 3:**
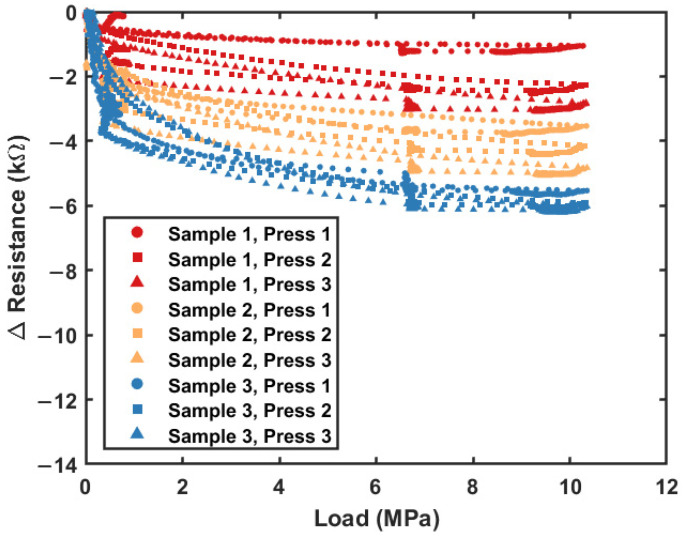
Piezoresistive response of LIG sensors in compression. Resistance change is shown for increasing load on three samples with three subsequent presses each.

To compare the recovery of LIG structures, the SEM images in Figure 2c,d (after pressing) can be compared to the images in Figure 1c,d (before pressing). Exact registration between SEM measurement locations is not possible, but minimal fiber breakage or pore collapse is seen in the images collected after pressing. Figure 3 shows the change in the resistance (ΔR) with the load during testing for three sensors pressed in triplicate. The load profile included: (1) constant displacement loading (0.25 mm/min) to 10.3 MPa, (2) hold for one minute, (3) constant displacement unloading (0.25 mm/min) to 6.9 MPa, (4) hold for one minute, and (5) constant displacement unloading (0.25 mm/min) to around 0.3 MPa. Triplicate presses did not remove the sensor from the test fixture between load cycles. Figure 3 shows three sensors (~5.2 J/cm^2^) printed on the same day in succession, and additional plots for different LIG laser fluences can be seen in Figure S4. 

Figure 1b and Figure 3 show that re-printing sensors had a more significant resistance change than replicate presses. Furthermore, data from Figure S4 show that re-printing sensors change resistance more than changing the recipes between prints. Despite the variation, each sample shows similar responses between successive presses, albeit with a slight downward trend. This indicates that although each sensor must be individually characterized for an initial response to ensure adequate sensitivity over the pressure range, the piezoresistive performance does not degrade significantly over time. 

### 3.3. Thermal Testing

**Figure 4:**
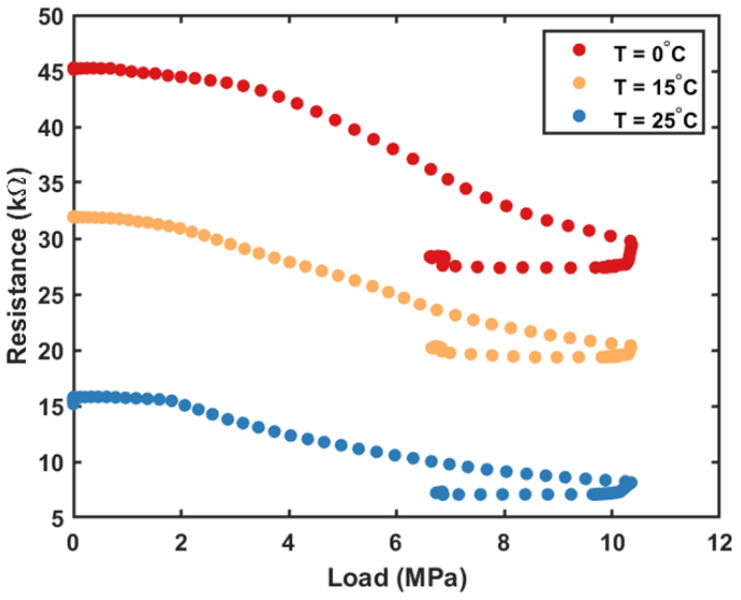
Piezoresistive response of LIG sensors with increasing load and decreasing temperature. Temperature changes are more significant than resistance changes.

Next, tests evaluated the piezoresistive response of LIG sensors with both compression and temperature (Figure 4). An MTS load frame performed the same load and unloading sequence as previously described, while a liquid nitrogen cooled environmental chamber replicated relevant ocean temperatures (0 to 25 °C) within 1 km of the surface. For this test, a Keithley SourceMeter was used to measure both the voltage and current over time. This test performed two successive presses after temperature equilibrium, and the second of each is shown in Figure 4.

Figure 4 confirms that there is also a strong thermal dependence on the LIG piezoresistive response. When comparing the magnitude of the thermal response and the pressure response, the resistance difference between different traces (temperatures) remains consistent at ~30 kΩ throughout the load profile, while the resistance difference between the initial and final loads (pressures) is between 5 and 15 kΩ depending on the temperature. This indicates that thermal insulation may be required to prevent error in depth measurements with temperature fluctuations.

**Figure 5:**
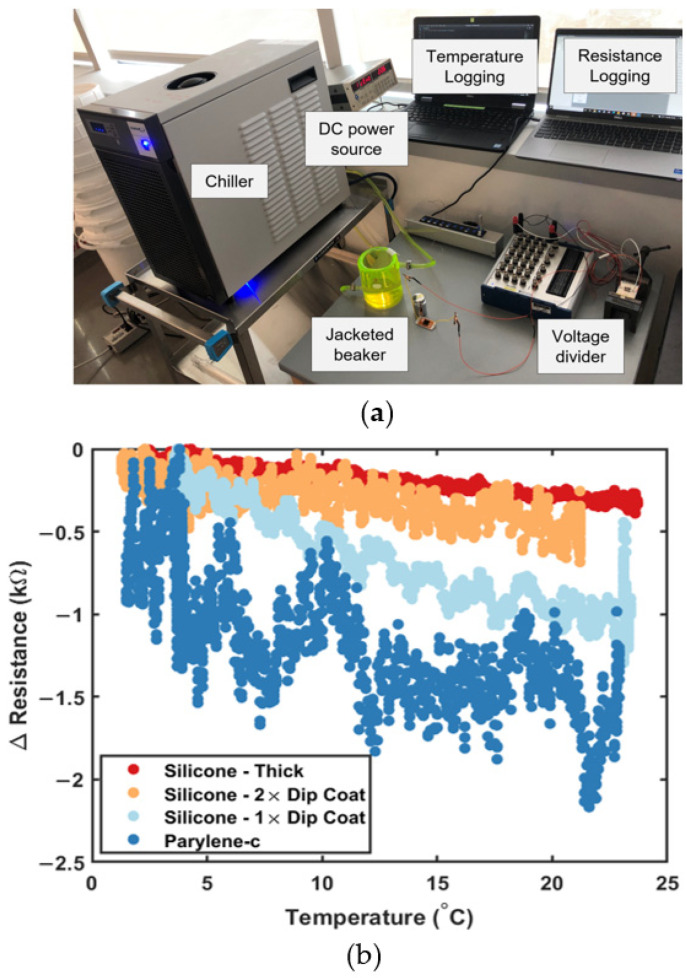
Thermal response of conformally coated LIG sensors shown with the (**a**) experimental setup with chiller, jacketed beaker, DC power source, voltage divider, and automatic temperature and resistance logging over time for (**b**) thick 10 wt% PDMS silicone layers, 2× dip coat of 5 wt% PDMS silicone, 1× dip coat of 5 wt% PDMS silicone, and vapor deposited 0.1 mm parylene-c layer.

Lastly, aqueous thermal tests with several conformal coatings simulated eventual marine deployment. Coatings serve to (1) electrically isolate resistive LIG traces from conductive seawater, (2) mechanically protect LIG traces from damage, and (3) thermally insulate LIG traces. Coatings tested included 5–10 wt% PDMS at different thicknesses (~1 cm, 2× dip coat, and 1× dip coat) chosen for its common use in marine applications and ease of fabrication, and parylene-c, an ultrathin (~0.1 mm) vapor-deposited coating, was chosen for its performance in aerospace environments, which exhibit similarly extreme pressures and cold temperatures. Variations in the PDMS silicone layer thickness investigated whether thicker and harder PDMS layers were necessary to prevent electrical shorting and thermally insulate traces given that the high compressive modulus of PDMS may dampen the LIG piezoresistive response. Figure 5a shows the thermal test experimental setup with a chiller and jacketed beaker to cool the interior water, DC power source and voltage divider to measure the resistive response (ΔR) of the LIG sensor, and temperature and resistance recording devices. Figure 5b shows the change in resistance over the tested temperature range (0 °C—room temperature).

All sensors show decreasing resistance in response to increasing temperature, with the ultrathin layer of parylene-c resulting in the largest resistance change (ΔR ≈ 1.2 kΩ), while the thick layer and 2× layers of dip-coated PDMS resulted in the smallest resistance changes (ΔR ≈ 0.2 and 0.3 kΩ, respectively). From these data, the 2× dip-coated PDMS layer is recommended for further testing to balance both improved thermal insulation and thin, low compressive modulus coatings for a good piezoresistive response. Durometer measurements of 5 wt% PDMS silicones had a Shore A hardness of 15, significantly softer than the manufacturer-recommended 10:1 base to curing agent mixing ratio (Shore A hardness = 44, compressive modulus ~ 1 MPa) [27,28]. Figure 5b also proves the necessity of conformal coatings for thermal insulation by demonstrating an approximately two orders of magnitude reduction in resistance change over the 0 to ~25 °C temperature range, allowing the LIG piezoresistive response to become the dominant effect.

## 4. Discussion

LIG fabrication, though facile, rapid, and inexpensive, depends on a complex combination of factors, resulting in high variability both within single prints and between distinct prints for the chosen parameters of this paper. A DOE approach attempted to pull out significant factors for predicting good LIG recipes, or laser fluences, that resulted in complete prints and more conductive (decreased resistance) traces. The analysis showed that though speed and power are necessary for predicting the completeness of prints, and have influence on predicting resistance, the most dominant factor is line length. The linear weighting of individual factors on the output response of the DOE indicates that creating larger LIG sensors should decrease the batch-to-batch piezoresistive response variability by both increasing the change in resistance with pressure above the measurement uncertainty and reducing the impact of laser speed and power on the LIG fabrication. 

Another key result indicated that the variation in LIG fabrication between batches is greater than the variation between the tested LIG recipes (speeds and powers). This can be seen in Figure 1b, where there is significant overlap of the error bars as laser fluence increases. The mean resistivities indicate that, in general, LIG resistivity decreases and becomes more conductive as laser fluence increases. The exception to this is the higher average resistivity values of the 5.2 J/cm^2^ recipe (average 1.40 ± 0.13 Ω·cm) compared to the 5.1 J/cm^2^ recipe (average 1.32 ± 0.11 Ω·cm) in Figure 1b. This deviance from the overall trend is most likely due to the non-linearity of the speed percentage, resulting in incorrectly approximated laser fluence values. The SEM images of graphene layers show that, in general, as laser fluence increases, larger, irregularly shaped, and more frequent pores form. Previous studies in the literature have shown that LIG films begin forming around laser fluences of ~5 J/cm^2^ [20], so potentially lower laser fluence values might result in more consistent porosity and predictable response. 

Similarly, the uniaxial compression characterization tests in this work indicated that the current LIG sensor fabrication pathway is not precise enough to produce replicate recipes, necessitating the individual characterization of each sensor before deployment. Given the rapid manufacturing time from polyimide sheet to full sensor (~30 min), in the future, LIG sensors could be prepared in bulk and be down-selected for the greatest resistance change in the target pressure range. However, after initial characterization, individual sensors performed similarly between replicate presses and recovered similar sensitivity between several cycles. Though the graphene formed is sensitive to abrasion, the porous structure recovers its original resistance quickly after compression. The slight downward trend with replicate presses is attributed to not allowing the sensors to fully relax in between tests (only unloading to ~0.3 MPa). Additionally, though LIG traces showed no visible breakage or deformation after six cycles, fatigue testing to a higher load and more cycles is needed to determine when LIG sensor failure occurs.

Lastly, all sensors show a decrease in resistance with increasing temperature, consistent with the negative temperature coefficient of carbon (−4.8 × 10^−4^ °C^−1^) [29]. In addition, Figure 4 indicates that bare LIG sensors have greater resistance change with temperature than pressure over the relevant marine temperature and pressure ranges, and any fielded system must integrate thermal insulation or temperature compensation to accurately measure depth. An ideal conformal coating for marine applications would have a low thermal conductivity to change its resistance only slightly with temperature fluctuations and be soft enough to not dampen the piezoresistive response. Therefore, the 2× dip-coated layers of 5 wt% PDMS silicone conformal coatings were selected for further testing (ΔR ≈ 0.3 kΩ) over thicker (~5 mm) and harder PDMS layers (ΔR ≈ 0.2 kΩ). PDMS readily wets all LIG recipes used in this work, forming interconnected porous structures of LIG/PDMS composites. This is consistent with the literature focused on the wettability of high-surface-area LIG structures formed under oxygen-rich (e.g., ambient air) atmospheres or at high laser fluence (surface oxygen content > ~5%) [23,30]. Encapsulating LIG sensors with liquid elastomer precursors (e.g., PDMS, EcoFlex, or polyurethane) is commonly used to fabricate flexible sensors to form LIG/elastomer composites [23]. Thin PDMS coatings (10 µm) have also been previously demonstrated to withstand up to 1.2 km simulated ocean depth without affecting thermistor sensor properties [19]. In this work, thinner layers (0.1 mm parylene-c and 1× dip-coated layer of 5 wt% PDMS silicone) did not provide adequate thermal insulation, resulting in a maximum resistance change of ~1.2 kΩ with parylene-c and ~1.0 kΩ with PDMS silicone. These values are similar to some of the measured resistance changes over the 10.3 MPa tested pressure range (see Figure S4), underlining the importance of temperature compensation approaches [31], or thicker PDMS silicone layers, for accurate resistance measurements in the field. 

As a rapidly emerging field, LIG sensors present exciting avenues for marine depth sensors. Soft and flexible pressure sensors remove the need for pressurized sensor suites at depth, which can greatly decrease the size and complexity of fielded systems [32]. Additionally, a fleet of rapidly manufactured sensors would allow for increased spatial and temporal resolution of the ocean and provide more data to protect vulnerable infrastructure or monitor the quickly changing environment. This work performed several experiments to evaluate using a commonly used industrial laser engraver to repeatably fabricate LIG piezoresistive depth sensors for large-volume deployment. Overall, choosing an appropriate laser engraver system with (1) high throughput capacity and (2) tunability for repeatable laser fluence is paramount. This would facilitate repeatable sensor fabrication without tedious post-assembly sensor characterization and potentially allow for targeted LIG porosities, fibrous surface morphologies, and wettability by controlling laser fluence. Additionally, this work determined that PDMS conformal coatings are essential for electrical isolation, mechanical stability, and thermal insulation. PDMS would also help prevent biofouling of the senor over time [19], which is known to be environmentally benign, and exhibits good chemical stability in water [28]. Further testing under hydrostatic pressure is needed to ensure LIG/PDMS composite sensors retain appropriate sensitivity over the pressure range. To reduce the compressive modulus, the PDMS curing agent weight percent can be further decreased, or other elastomers can be substituted (e.g., polymethyl methacrylate (PMMA) or thermoplastic polyurethane (TPU) previously proposed for marine depth sensors) [33,34]. LIGs for marine applications are also not limited to depth sensing. Previous research has proposed LIG salinity and temperature sensors for full suite CTD measurements [19], velocity sensors as low-power marine mammal aquatic tags or coral reef current measurements [35], and even force sensors of diving equipment or soft robotic manipulators [32]. Additionally, design modifications of LIG, or other flexible piezoresistive composite structures, have been proposed that could improve performance in marine environments. Through a DOE, this work indicated that a more reliable piezoresistive response could be achieved with larger sensors. This would have to be balanced by larger voltage requirements to power longer line lengths, and increased joule heating would be needed. Alternatively, trace conductivity can be increased and input voltages decreased by depositing conductive particles on LIG traces such as eutectic gallium indium (EGaIn) [36]. Similarly, alternative materials such as recently proposed gold/chromium (Au/Cr) serpentine traces made via photolithography can be leveraged for piezoresistive depth sensors [32]. Resistive traces can also be changed to radially symmetric shapes to reduce the strain distribution under a high hydrostatic load and improve the pressure response [32]. Researchers could also investigate different capacitive sensing mechanisms where dual electrodes are separated by a compressive dielectric layer, as demonstrated with porous PDMS depth sensors sandwiched by aluminum-coated polymer electrodes [28]. Overall, researchers have identified a need for low-cost and low-power marine sensors to improve monitoring capabilities, and LIG sensors and composite systems are an evolving field that could address these challenges.

## 5. Conclusions

This work investigated using an industrial laser engraver to characterize LIG pressure sensor fabrication parameters and piezoresistive response over relevant temperature and pressure ranges for a marine depth sensor. Initially, a DOE identified four LIG recipes with laser fluences ranging from 4.6 to 7.2 J/cm^2^ and determined that line length was the most significant factor on resistivity. During uniaxial compression testing, sufficient repeatability between replicate prints was not achieved, though LIG sensors showed a measurable response over the relevant 1 km depth pressure range. This indicated that individual sensor calibration or alternative laser engravers for improved control over laser fluence are needed. Additionally, over the four selected recipes, unencapsulated LIG sensors performed better as thermistors compared to pressure sensors, exhibiting a 30 kΩ resistance change from 0 to 25 °C, and between 1 and 15 kΩ resistance change from 0 to 10.3 MPa (~1 km ocean depth). This thermal response was reduced significantly (100× to 0.3 kΩ) with the addition of two dip-coated layers of a lower compressive modulus (5 wt% curing agent) PDMS silicone conformal coating. However, concerns about decreased compressive performance with porous PDMS/LIG composite structures reducing compressible area, and thus sensitivity in hydrostatic testing, must be addressed in the future. Overall, this work highlights that the large-scale fabrication and deployment of LIG piezoresistive depth sensors is a non-trivial task that requires additional engineering efforts to facilitate. However, the rapidly advancing field is poised to meet these material challenges and forge a path toward distributed networks of rapidly manufactured LIG sensors. 

## Figures and Tables

**Table 1:** LIG recipes determined via a DOE.

Name	Power (%) ^1^	Speed (%) ^2^	Approximate Laser Fluence (J/cm^2^)
S65P45	45	65	4.6
S65P50	50	65	5.1
S70P55	55	70	5.2
S55P60	60	55	7.2

^1^ Percentage of 50W CO_2_ laser (linear). ^2^ Percentage of 4.2 m/s (165 in/s) maximum laser speed [26].

## Data Availability

The data presented in this study are available on request from the corresponding author.

## Supplementary Materials

The following supporting information can be downloaded at: https://www.mdpi.com/article/10.3390/s23229044/s1, Figure S1: Operational limits of laser printer; Table S1: Operational limits of laser printer; Figure S2: DOE analysis for print parameters; Table S2: Resistance values for DOE; Figure S3: Additional SEM images; Figure S4: Additional Instron testing.
