# Supplementary material for: Practical Considerations for Laser-Induced Graphene Pressure Sensors Used in Marine Applications
Source: Sensors (Basel). 2023 Nov 8;23(22):9044. doi: 10.3390/s23229044 (PMC10674168; doi:10.3390/s23229044)
Supplement: Supplementary file 1 [file sensors-23-09044-s001.zip › sensors-2665826-supplementary.pdf]

sensors

MDPI

Supplemental Data

Practical Considerations for Laser Induced Graphene Marine Depth Sensors

Tessa Van Volkenburg, Daniel Ayoub, Andrea Alemán Reyes, Zhiyong Xia, and Leslie Hamilton*

*Correspondence: Leslie.Hamilton@jhuapl.edu

1. Operational Limits of Laser Printer

Initially, the minimum line width and spacing the laser engraver could repeatedly print was established through the templates shown in Figure S1a. The resultant laser induced graphene (LIG) prints are shown in Figure S1b and the good parameters tested are highlighted in Table S1.

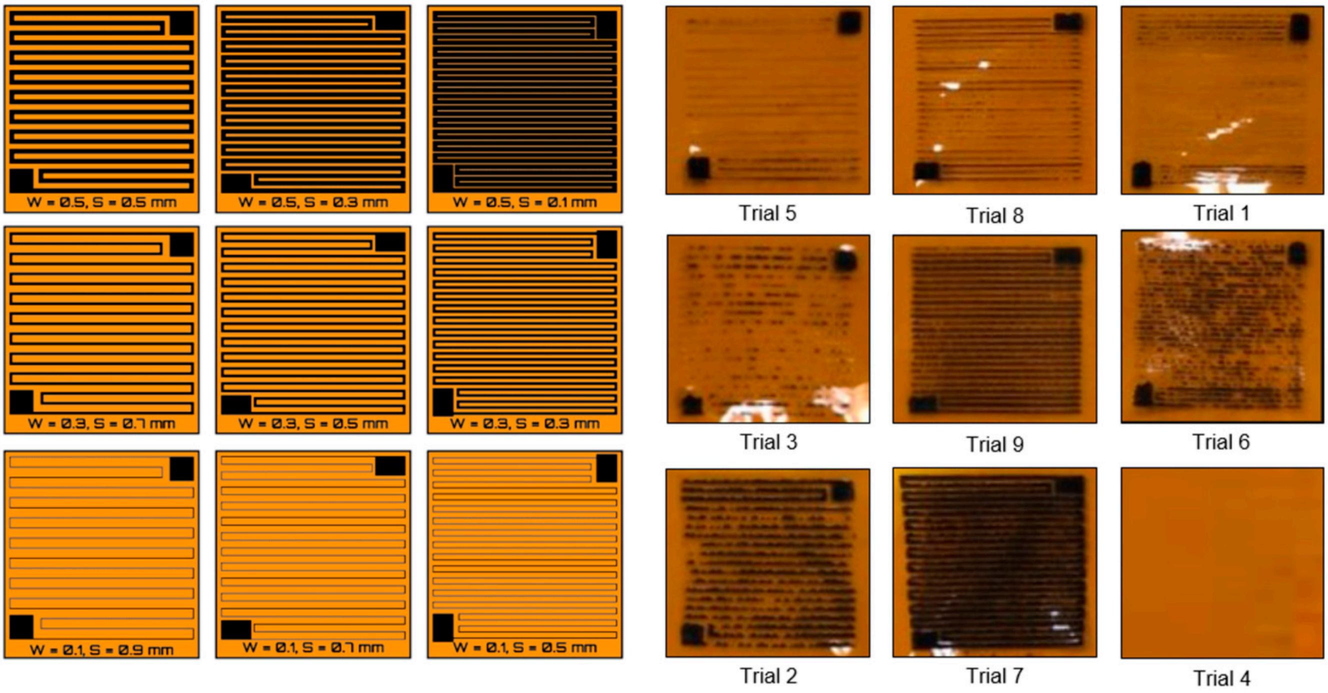

(a) (b)

Figure S1. Prints to determine the operational limits of the laser printer with a) template and b) actual prints.

Table S1. Operational limits of the laser printer.

Trial	Pattern	Power (%)	Speed (%)	Line Width (mm)	Line Spacing (mm)
1	3	45	70	0.1	0.5
2	7	45	75	0.5	0.9
3	4	45	80	0.3	0.9
4	9	50	70	0.5	0.5
5	1	50	75	0.1	0.9
6	6	50	80	0.3	0.5
7	8	55	70	0.5	0.7
8	2	55	75	0.1	0.7
9	5	55	80	0.3	0.7

[1] Highlights indicate good prints

2. DOE Analysis for Print Parameters

Design of experiment (DOE) pattern and prints are shown in Figure S2a, and the resultant John Macintosh's Project (JMP) statistical analysis software leverage plot and significant factors are shown in Figure S2b. Additionally, Table S2 lists the average resistance measured across the contact patches where incomplete prints are marked with a dash and omitted from the analysis. The actual vs. predicted, or "leverage plot" in Figure S2b compares the null hypothesis (blue horizontal line, assumes response is independent of the factors) to the alternative hypothesis (red slanted line, assumes response depends on the factors). The shaded red region indicates the 95% confidence intervals. The low R^2 value (0.44) indicates the model fit line is not sufficient to describe the dataset, though the overall trend in resistance can be reasonably inferred from the low PValue. The individual and combinatorial significant factors (PValue < 0.05) are also listed by their relative importance.

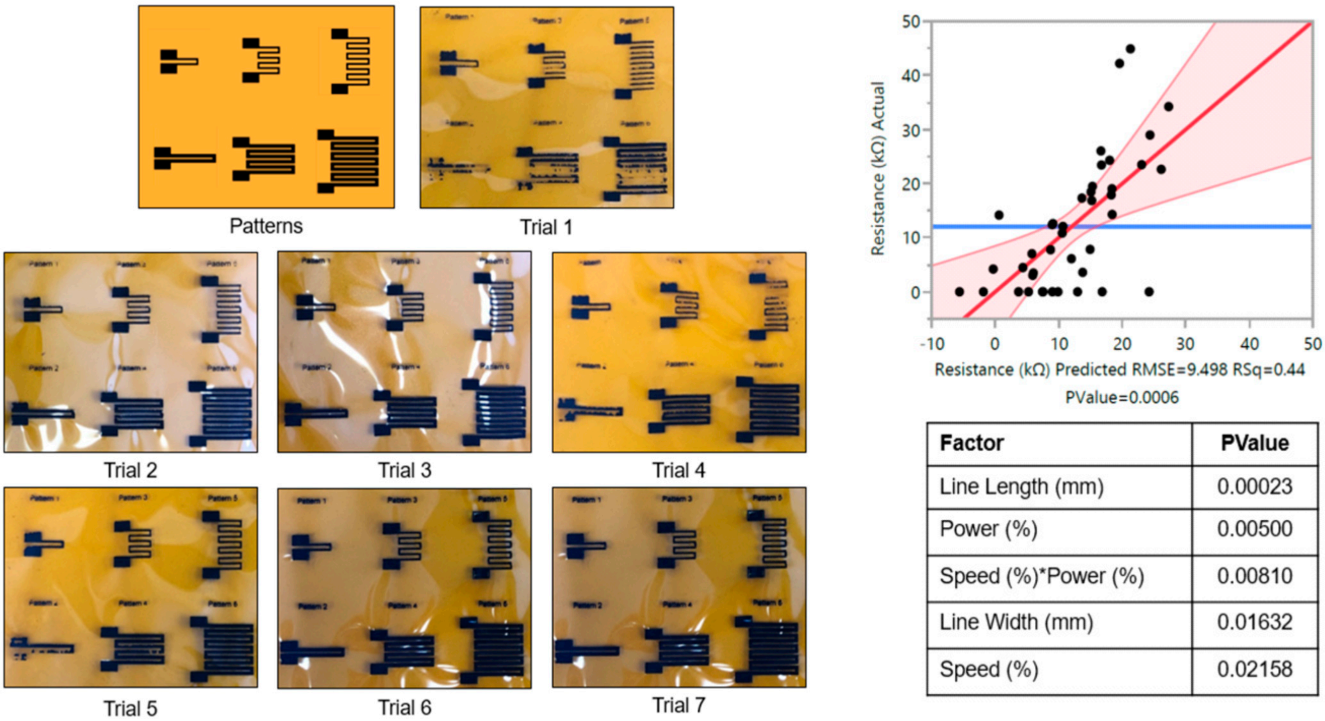

(a) (b)

Figure S2. DOE analysis a) print trials, b) expected vs. predicted values with significant factors.

Table S2. Resistance values for DOE.

Pattern	Trial 1 S70P50	Trial 2 S70P55	Trial 3 S65P55	Trial 4 S60P50	Trial 5 S60P60	Trial 6 S55P65	Trial 7 S55P60
1	14.2	7.8	6.1	4.5	3.6	3.1	3.5
2	-	7.8	7.0	-	-	4.2	4.2
3	-	42.2	26.0	-	14.3	10.9	12.1
4	-	24.3	18.5	17.3	-	12.4	12.6
5	-	-	44.9	-	23.5	16.9	19.5
6	-	34.2	29.0	23.4	22.6	17.9	19.0

3. Repeatability of Measurements

Figure S3 shows scanning electron microscope (SEM) images of LIG surfaces for the intermediate LIG recipes. Here again, higher laser fluences result in larger, more varied pores, and more fibrous branching regions on the surface.

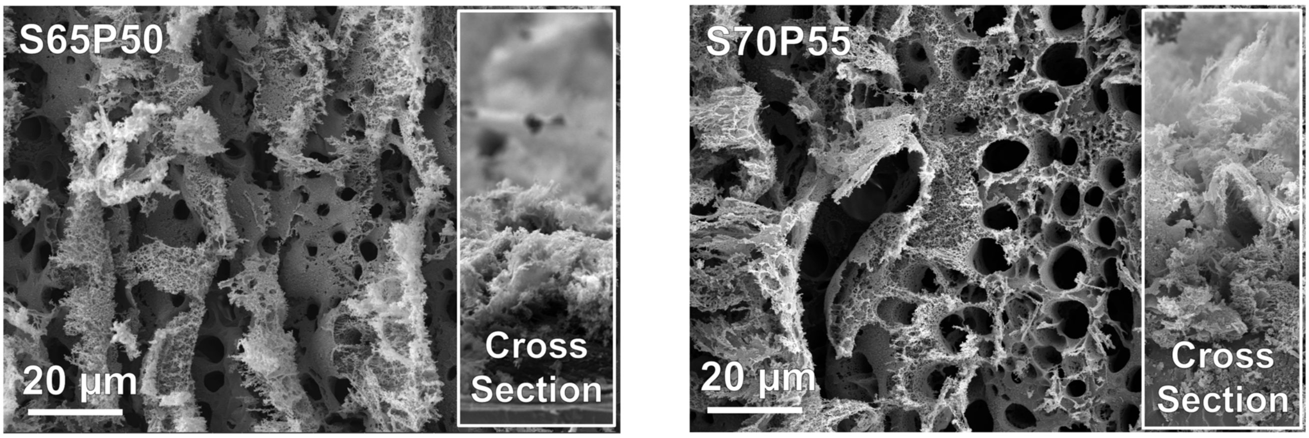

(**a**) (**b**)

Figure S3. Additional SEM images of LIG laser recipes for a) 5.1 J/cm^2 and b) 5.2 J/cm^2. Porosity and fibers seen with top-down and cross-sectional (inset) views.

Figure S4 shows additional laser fluence recipes and their response to uniaxial compression testing. Again these tests show that batch to batch variability dominates over the chosen laser parameters, necessitating individual LIG sensor characterization. Good recovery and repeatability of triplicate presses of the same sensor is also seen.

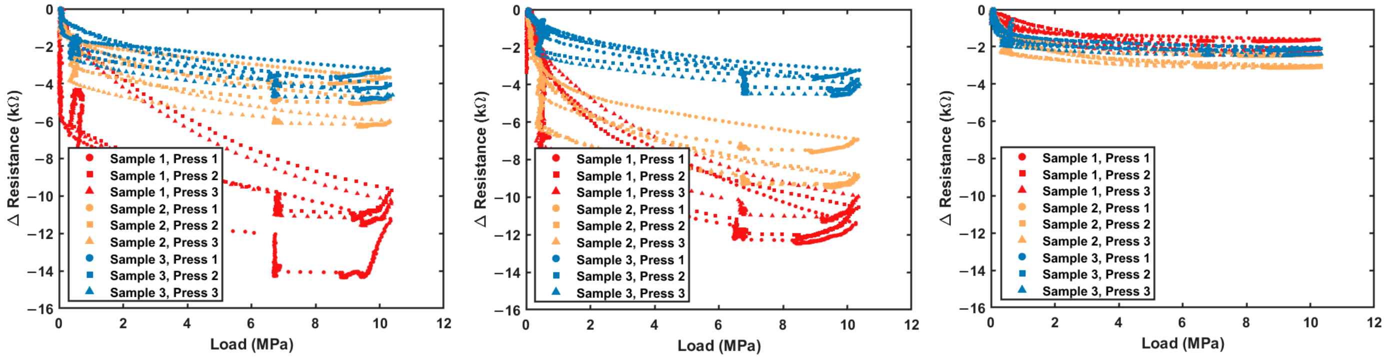

(**a**) (**b**) (**c**)

Figure S4. Additional Instron testing of different LIG laser recipes for a) 4.6 J/cm^2, b) 5.1 J/cm^2, and c) 7.2 J/cm^2 laser fluences. Colors indicate same samples, and shapes indicate subsequent presses. Greater variability is seen between recipes than load profile.
